# Stratification of Tamoxifen Synergistic Combinations for the Treatment of ER+ Breast Cancer

**DOI:** 10.3390/cancers15123179

**Published:** 2023-06-14

**Authors:** Emily K. Zboril, Jacqueline M. Grible, David C. Boyd, Nicole S. Hairr, Tess J. Leftwich, Madelyn F. Esquivel, Alex K. Duong, Scott A. Turner, Andrea Ferreira-Gonzalez, Amy L. Olex, Carol A. Sartorius, Mikhail G. Dozmorov, J. Chuck Harrell

**Affiliations:** 1 Department of Pathology, Virginia Commonwealth University, Richmond, VA 23298, USA; zborilek@vcu.edu (E.K.Z.);; 2 Department of Biochemistry and Molecular Biology, Virginia Commonwealth University, Richmond, VA 23298, USA; 3 Integrative Life Sciences Program, Virginia Commonwealth University, Richmond, VA 23298, USA; 4 C. Kenneth and Dianne Wright Center for Clinical and Translational Research, Virginia Commonwealth University, Richmond, VA 23298, USA; 5 Department of Pathology, University of Colorado Anschutz Medical Campus, Aurora, CO 80045, USA; 6 Department of Biostatistics, Virginia Commonwealth University, Richmond, VA 23298, USA; 7 Massey Cancer Center, Virginia Commonwealth University, Richmond, VA 23298, USA; 8 Center for Pharmaceutical Engineering, Virginia Commonwealth University, Richmond, VA 23298, USA

**Keywords:** estrogen receptor, ER+ breast cancer, tamoxifen, endocrine resistance, drug synergism, patient-derived xenograft

## Abstract

**Simple Summary:**

ER+ breast cancer is the most diagnosed subtype and patient prognosis has improved in recent years thanks largely to the development of endocrine-targeting therapies including tamoxifen. Unfortunately, many tumors will recur as endocrine-therapy-resistant metastases. Therefore, in order to prolong resistance-free survival, combination therapies that mitigate resistance mechanisms should be pursued. In this study, we tested 516 drug combinations with tamoxifen and identified two that were synergistic. These combinations inhibited breast cancer patient-derived xenograft (PDX) growth in animal models better than either drug or tamoxifen alone.

**Abstract:**

Breast cancer alone accounts for the majority of cancer deaths among women, with the most commonly diagnosed subtype being estrogen receptor positive (ER+). Survival has greatly improved for patients with ER+ breast cancer, due in part to the development of antiestrogen compounds, such as tamoxifen. While treatment of the primary disease is often successful, as many as 30% of patients will experience recurrence and metastasis, mainly due to developed endocrine therapy resistance. In this study, we discovered two tamoxifen combination therapies, with simeprevir and VX-680, that reduce the tumor burden in animal models of ER+ breast cancer more than either compound or tamoxifen alone. Additionally, these tamoxifen combinations reduced the expression of HER2, a hallmark of tamoxifen treatment, which can facilitate acquisition of a treatment-resistant phenotype. These combinations could provide clinical benefit by potentiating tamoxifen treatment in ER+ breast cancer.

## 1. Introduction

### 1.1. ER+ Breast Cancer and Standard of Care

Breast cancer is the most commonly diagnosed cancer and leading cause of cancer death among women [1]. Breast tumors can be classified by (1) the expression of three receptors, namely, the estrogen receptor (ER), progesterone receptor (PR), and human epidermal growth factor receptor 2 (HER2) and (2) differential transcriptomic signatures such as the PAM50, which, among additional clinical biomarkers, are used to parse breast cancers into one of five intrinsic molecular subtypes (luminal A, luminal B, HER2-enriched, basal-like, and normal-like) [2,3]. ER+ breast cancers are stratified into either the luminal A or B subtype, for which proliferative status and PR expression are differentiating factors. Approximately 70–80% of breast cancers are ER+ at diagnosis and are largely driven by estrogenic activity [4].

In addition to chemotherapy, radiation, and surgical resection, current standard of care regimens for women with ER+ breast cancer focus on the reduction in circulating estrogen or antagonizing ER [5]. These adjuvant endocrine therapy options include selective estrogen-receptor receptor modulators (SERM), the most common of which is tamoxifen, as well as aromatase inhibitors (AI), including letrozole, anastrozole, and exemestane, which block estrogen production [6]. Data from randomized clinical trials suggest five years of adjuvant endocrine therapy reduce recurrence by 50%, and extending treatment to 10 years further improves disease-free survival even beyond the cessation of treatment [7]. AIs are generally prescribed for post-menopausal women, as considerable amounts of estrogen precursors in the ovary limit the therapeutic potential of these compounds in pre-menopausal patients [8]. Tamoxifen is prescribed for pre-menopausal women and any post-menopausal women who may be unable to take AIs due to contraindications or adverse effects [9]. Other treatment options include selective ER degraders (SERD) such as fulvestrant, and oophorectomy. More recently, CDK4/6 inhibitors including palbociclib, ribociclib, and abemaciclib, as well as PI3K targeting compounds, have been introduced to first- and second-line therapy [5,10].

### 1.2. Etiology of Endocrine Resistance

Though often initially clinically responsive, approximately 30% of patients with ER+ disease will develop long-term endocrine resistance [11]. Considering the loss of ER expression accounts for only 10% of endocrine-resistant breast cancers, understanding the different modalities of the emergence of therapeutic resistance is critical [12]. Much work has been carried out to understand the molecular underpinnings of tamoxifen resistance specifically. Tamoxifen resistance can be the result of direct modulation of ER signaling, the upregulation of receptor tyrosine kinases (HER2, EGFR, FGFR, etc.), aberrations in the PI3K/AKT/mTOR pathway, or activation of NFkB signaling [13]. Targeting these pathways in addition to ER could increase durability, or extend progression-free survival, in patients with ER+ disease, as well as decreasing endocrine therapy resistance.

To overcome resistance, or to potentiate antiestrogen therapies, combination regimens are often prescribed [14]. Many clinical studies evaluating tamoxifen in combination with other compounds and treatments have been performed. For example, trials pairing tamoxifen with EGFR inhibitors (NCT04504331) have been conducted. Additionally, evaluating gonadotropin-releasing hormone agonists (NCT00066703) or the suppression of ovarian function (NCT00066690) with tamoxifen have been performed. As tamoxifen has been used in the treatment of other hormone-sensitive cancers, additional trials have tested the efficacy of tamoxifen with SUBA-Itraconzole, an anti-fungal medication, for the treatment of ovarian cancer (NCT05156892).

### 1.3. PDX Models/Models of ER+ BC

Patient-derived xenograft (PDX) models are established by directly implanting human tumor cells orthotopically into immune-deficient mice [15]. PDX models are useful in studying translational science, as they faithfully recapitulate facets of human disease such as tumor heterogeneity, therapeutic response, and genomic evolution [16,17]. For these reasons, in vivo and ex vivo PDX models are a useful complement to cell lines [18,19].

### 1.4. Approach

To overcome drug resistance and increase treatment durability, tamoxifen combination therapies may be pursued. Because novel approaches are necessary, we utilize high-throughput screening (HTS) to discover therapeutic combinations with tamoxifen. To ensure transferability, the combinations that reduced cell viability to the greatest degree were further evaluated, and two were tested in vivo.

We show that tamoxifen and either VX-680, an aurora kinase inhibitor, or simeprevir, a hepatitis C antiviral compound, are synergistic in PDX models of ER+ breast cancer. Additionally, both combinations effectively reduce tumor volume in vivo, more than either compound or tamoxifen alone. We illustrate the capability of these tamoxifen combination therapies to suppress the upregulation of HER2, which can facilitate endocrine resistance. These results highlight the importance of addressing tamoxifen resistance mechanisms in the form of primary treatment to garner a more durable response.

## 2. Materials and Methods

### 2.1. Patient-Derived Xenograft (PDX) Models

HCI-011 and HCI-013 were obtained from the Huntsman Cancer Institute, University of Utah; BCM-5097, BCM-15057, and BCM-15034 were obtained from Baylor College of Medicine, Houston, TX, USA. All mouse studies were performed in accordance with the Virginia Commonwealth University (VCU) Institutional Animal Care and Use Committee (IACUC). Xenografts were grown in the fourth mammary fat pad set of 5–7-week-old female non-obese diabetic severe combined immunodeficient gamma (NSG) mice (NOD.Cg-Prkdcscid Il2rgtm1Wjl/SzJ, JAX #005557) [20,21]. Tumor digestion was carried out as previously described. Briefly, tumors were harvested once approximately 300 mm^3^, finely minced, and digested for one hour at 37 °C with agitation in DMEM/F12 supplemented with 5% FBS, 300 U/mL collagenase (Sigma, St. Louis, MO, USA), and 100 U/mL hyaluronidase (Sigma, St. Louis, MO, USA). Red blood cells were lysed and a single-cell suspension was achieved through trypsin digestion. Cells were resuspended in Hanks’ balanced salt solution (HBSS) with 2% FBS for downstream applications. For in vivo expansion and propagation of PDXs, tumor cells were suspended 1:1 in Cultrex (Bio-Teche, Minneapolis, MN, USA) and injected bilaterally into the fourth set of mammary glands of NSG mice at 500,000 cells per injection. Silastic estrogen pellets (2 mg) were implanted subcutaneously at the time of injection to supplement exogenous estrogen [22]. Tumor growth was monitored longitudinally by weekly caliper measurements.

#### Development of Endocrine-Resistant PDX Models

For generation of the BCM-15057EI variant, cells were grown as orthotopic tumors in intact female NSG mice with supplemental estrogen via subcutaneous silastic pellet. Once tumors reached approximately 50 mm^3^, the subcutaneous estrogen pellet was surgically removed. Following tumor regression and subsequent resurgence, tumors were harvested and passaged into ovary-intact mice without exogenous estrogen. Tumors were harvested when they reached 300 mm^3^ and passaged into ovariectomized mice without supplemental estrogen, wherein tumor growth was observed, and the subline was deemed estrogen independent (EI). To generate a tamoxifen-resistant PDX, BCM-15034 cells were grown orthotopically with supplemental estrogen. Once tumor burden reached 50 mm^3^, mice were transferred to tamoxifen citrate chow (Envigo, Indianapolis, IN, USA, 600 mg/kg). Tumors were then serially passaged into mice and grown in the presence of tamoxifen, until no difference in growth rate was observed comparing tumors grown under standard breeder chow versus tamoxifen-supplemented chow, wherein this isogenic line was deemed tamoxifen-resistant (BCM-15034TamR).

### 2.2. Cell Lines

Human ER+ breast cancer cell lines MCF7 and T47D were obtained from the American Type Culture Collection (ATCC, Manassas, VA, USA), and cultured in RPMI-1640 supplemented with GlutaMAX, 10% fetal bovine serum (FBS), and penicillin/streptomycin. UCD12 cells were a generous gift from Carol Sartorius, Ph.D. (University of Colorado Anschutz Medical Campus, Aurora, CO 80045 USA). UCD12 cells were cultured in Dulbecco’s modified minimum essential medium (DMEM)/F12 containing L-glutamine (365  mg/L) buffered with sodium bicarbonate (1200  mg/L) and HEPES (3575  mg/L) with 10% FBS, cholera toxin (100  ng/mL), hydrocortisone (1  μg/mL), insulin (10^−9^ M), and penicillin/streptomycin. All cell lines were maintained at 37  °C with 5% CO_2_.

### 2.3. Bulk RNA Sequencing

#### 2.3.1. Bulk RNA Sequencing Analysis

RNA was isolated using Qiagen RNeasy and QIAshredder kits according to the manufacturer’s protocol from flash-frozen tumor fragments. Quality of RNA was assessed using TapeStation (Agilent Technologies, Santa Clara, CA, USA). Library preparation was performed using the NEBNext Ultra II RNA Library Prep Kit for Illumina (New England Biolabs, Ipswich, MA, USA) according to the manufacturer’s instructions. Oligod(T) beads were then used to enrich for mRNA, from which cDNA libraries were generated. After end repair, universal adapters were ligated via PCR with limited cycles. Library validation and quality control were performed by TapeStation and qPCR (KAPA Biosystems, Wilmington, MA, USA).

Illumina HiSeq 4000 was used to perform 2 × 150 bp paired-end sequencing, according to instrument protocols by Azenta Life Sciences. Base calling and image analysis were conducted using HiSeq Control Software (HCS) v3.3.2-v3.4.0. Raw BCL data were converted to FASTQ file format and demultiplexed with Illumina BCL2FASTQ 2.17. One mismatch was allowed for index sequence identification.

#### 2.3.2. Quality Control and Pre-Processing

Preprocessing of bulk RNA-seq data has been described previously [23,24]. Briefly, assessment of sequencing quality was conducted using FastQC v0.11.8 [25]. Adapters and low-quality base pairs were removed using CutAdapt v1.15 [26]. Reads of sufficient quality were aligned using STAR v2.5.2b to a merged human/mouse genome using the command line options: “--outSAMtype BAM Unsorted --outSAMorder Paired --outReadsUnmapped Fastx --quantMode TranscriptomeSAM --outFilterMultimapNmax 1. The Salmon v0.8.2; “quant” algorithm was used to obtain read counts from the aligned BAM files using the “IU” library type [27,28]. For details on merged genome construction, see Alzubi et al. [24]. Log2TPM values were calculated in R and used for gene signature computations and PAM50 subtyping was carried out using genefu v2.11.2 R package [29]. New and previously generated data were included in this study (Supplementary Table S1).

#### 2.3.3. Gene Signatures and Clustering

Previously published gene expression signatures were used to generate signature scores for each PDX by averaging bulk RNA-seq Log2 TPM values from genes included in from each signature [30,31,32,33,34,35,36,37,38,39,40,41,42,43,44]. Unsupervised hierarchical clustering was performed in Morpheus using one minus Pearson’s correlation as the distance metric to cluster both rows and columns of the generated gene signature score matrix.

### 2.4. Targeted Mutational Profiling

Oncomine Comprehensive Assay v3 was performed as described [45]. Briefly, PDX tumor fragments were flash frozen and embedded in Optimal Cutting Temperature (OCT) compound prior to nucleic acid isolation. Next-generation sequencing (NGS) in accordance with Oncomine Comprehensive Assay v3 (Thermo Fisher, Waltham, MA, USA) was performed by the VCU Pathology Molecular Diagnostics laboratory with clinically validated methodology used for diagnostic tumor profiling.

### 2.5. Single-Cell RNA Sequencing

#### Single-Cell RNA Sequencing Quality Control and Preprocessing

Single-cell RNA sequencing (scRNA-seq) was performed on five ER+ breast cancer PDXs (BCM-5097, BCM-15057, BCM-15034, HCI-011, and HCI-013) that were grown orthotopically in NSG mice that were fed normal breeder chow, or in some cases tamoxifen citrate chow. Tumors were dissociated as described previously, and cells were prepared using the Chromium singles cell gene expression kit (10x Genomics, Pleasanton, CA, USA), according to the manufacturer’s instructions. Sequencing was performed at the VCU Genomics core. Reads were aligned to the 10x Genomics human/mouse genome (human being GRCh38, and mouse mm10) in order to remove reads from mouse cells. The remaining human cells were realigned to the human genome, and dead/poor-quality cell filtering was performed using an in-house R v4.1.3 script with the Seurat v.4.3.0 package as described in Boyd et al. [18]. Samples were then normalized using log normalization and merged using Seurat’s merge () function. The merged data were saved as a 10xcounts “.h5” file with all cell annotations saved as .CSV files. The CellRanger “reanalyze” function was then employed to convert the H5 file to a “.cloupe” file so that it could be uploaded Into the 10x Loupe browser for exploration.

### 2.6. Drug Screening

High-throughput screening was performed on PDX and cell line models of ER+ breast cancer using a 516-drug library at 1 μM concentration, as reported previously [46]. Two biological replicates were averaged, and coefficient of drug interaction (CDI) was calculated for each drug in each model, using the formula CDI = AB/(A × B), where A and B are the viability of tamoxifen and each agent alone, and AB is the viability of the combination [23].

#### 2.6.1. In Vitro

In vitro drug screening of PDX cells has been previously described [23,46,47]. In brief, single-cell suspensions were plated in 96-well plates at a density of 25,000 cells/well in M87 medium and treated for 72 h. For cell line viability assays, 1250 cells per well were plated in 96-well plates in RPMI-1640 supplemented with GlutaMAX, 10% FBS, and penicillin/streptomycin and allowed to adhere for 24 h. Cells were treated for 72 h, and viability was measured relative to vehicle-treated control using the CellTiter-Glo Luminescent Viability Assay (Promega, Madison, WI, USA), according to the manufacturer’s protocol. For 4-hydroxytamoxifen synergism screens, after cells had adhered, media were removed and replaced with RPMI + GlutaMAX with 10% FBS, 1% penicillin/streptomycin, and 4-hydroxytamoxifen at the cell line-specific IC20 dose. Cells were then administered drugs and viability was determined as described above.

#### 2.6.2. In Vivo

Pilot studies were performed to determine tolerable doses of simeprevir (MedChem Express, Junction, NJ, USA, HY-10241) and VX-680 (MedChem Express, HY-10161). For in vivo experiments, tumors were seeded, and, once palpable, mice were randomized into respective treatment groups. VX-680 (70 mg/kg) and simeprevir (40 mg/kg) were suspended in sterile saline and PEG300 at a 50:50 ratio. Doses were administered 5 days per week via intraperitoneal (IP) injection. Tamoxifen treatment was administered via chow (Envigo, 600 mg/kg). Tumors were measured 3 times per week, and tumor area was calculated using the formula A = L/W.

### 2.7. Western Blot

Tumor fragments were homogenized in a buffer containing 8 M Urea, 1% SDS, 200 mM EPPS pH 8.5 with protease and phosphatase inhibitors. Protein electrophoresis and transfer were performed using BIO-RAD PowerPac Basic and BIO-RAD Tran-Blot Turbo, respectively. Immunoblotting was performed using antibodies against ER (Abcam ab16660, Cambridge, UK), TFF1 (Cell Signaling #15571, Danvers, MA, USA) and GAPDH (Cell Signaling #5174). Detection was achieved using LI-COR Odyssey Fc (987-15227) with Image Studio v5.2.

### 2.8. Immunohistochemistry (IHC)

PDX tissues were processed and embedded by the Tissue and Data Acquisition and Analysis Core (TDAAC) at VCU. Antigen retrieval was performed using EDTA/TRIS Antigen retrieval buffer in a decloaking chamber (DakoCytomation, Glostrup, Denmark, Pascal). Immunohistochemical staining was performed using antibodies against ER (Abcam ab16660), PR (Cell Signaling #8757), HER2 (Cell Signaling #2242), Ki67 (Cell Signaling #9027), aurora kinase A (Cell Signaling #91590), and aurora kinase B (Cell Signaling #28711). HRP-conjugated anti-rabbit secondary binding was detected using DAB and images were acquired using Zen 2 (blue edition) software.

### 2.9. Quantification and Statistical Analysis

Densitometry of western blot experiments was quantified using Empiria Studio^®^ Software v2.2 (LI-COR, Lincoln, NE, USA). Quantification of IHC was conducted using Fiji (ImageJ 2.9.0). All statistical analysis was performed using GraphPad Prism version 9.

## 3. Results

### 3.1. Tamoxifen Treatment Response Was Classified in Five ER+ PDX Models In Vivo

**Figure 1:**
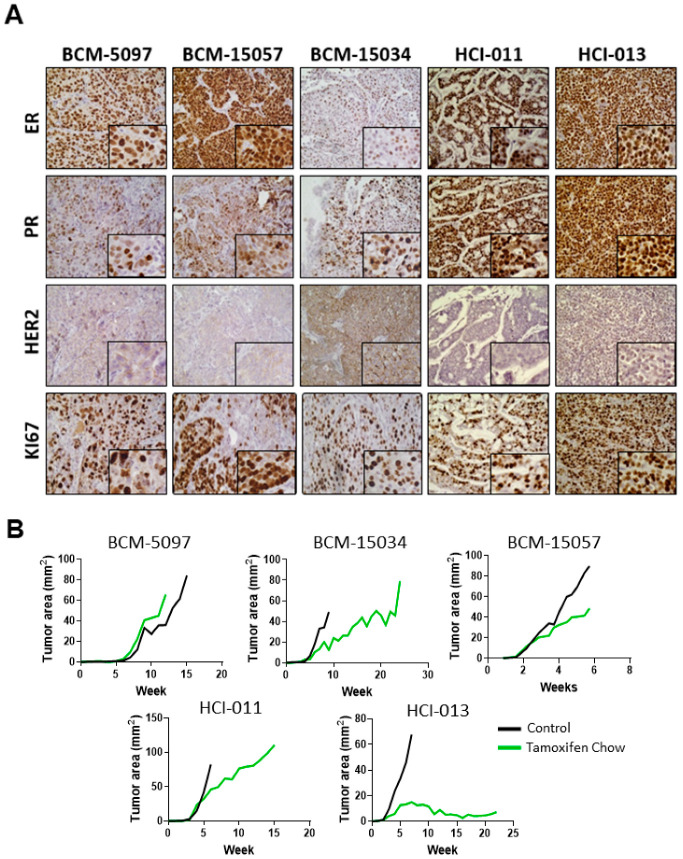
Hormone receptor expression in ER+ PDX models and response to tamoxifen treatment. (**A**) Representative immunohistochemical staining of ER, PR, HER2, and Ki67 in 5 ER+ PDX mammary gland tumor models with high magnification insets. Original images were collected at 400× magnification. (**B**) Growth curves of 5 ER+ PDX mammary tumors in untreated and tamoxifen-treated groups.

Histological analysis of five ER+ PDX models was performed to confirm the expression of hormone receptors, HER2, and the proliferation marker Ki67. Additionally, in vivo dose response curves were collected to establish sensitivity to tamoxifen in ER+ breast cancer PDX models. Analysis of the five ER+ PDX models showed varying expressions of ER and PR. HCI-011 and HCI-013 highly expressed ER and PR, as well as Ki67; however, HCI-013 was extremely responsive to tamoxifen treatment, with the tumor burden being reduced by more than half. Conversely, BCM-5097 was intrinsically resistant to tamoxifen, with the tumor burden being comparable between tamoxifen-treated and untreated groups. HER2 expression was most evident in BCM-15034, which is expected, as this model was generated from a patient with HER2-amplified disease (Figure 1A, Supplementary Figures S1 and S2). Therefore, this model is considered triple positive (ER+ PR+ HER2 amplified) [48]. In all other models interrogated, tumor growth was delayed compared to control; however, tamoxifen treatment did not decrease BCM-15057, BCM-15035 or HCI-011 tumor size at any point (Figure 1B).

### 3.2. Tumor Heterogeneity Is Conserved in PDX Models

**Figure 2:**
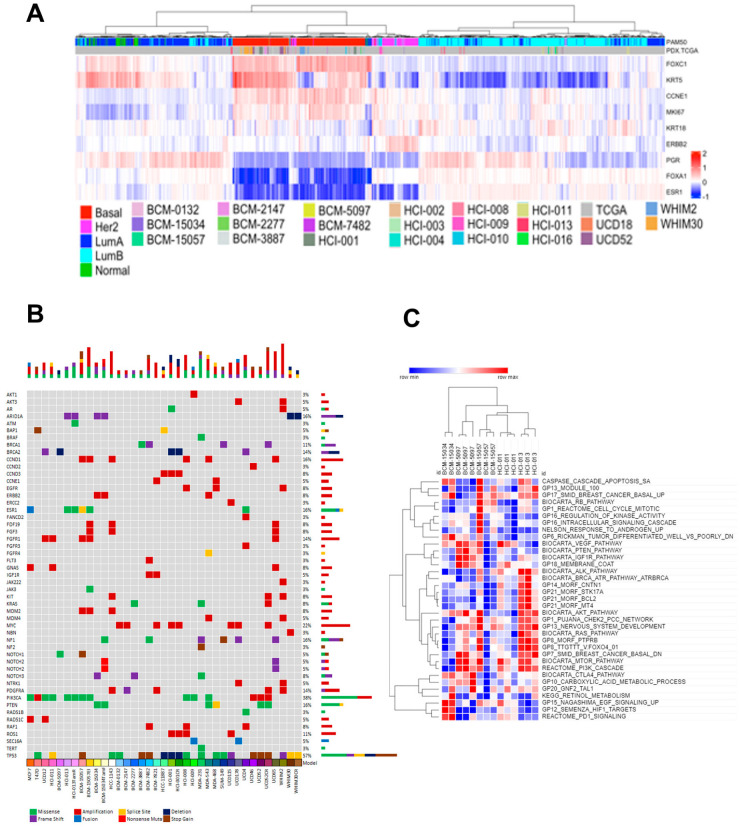
Transcriptomic and genomic profiling of a bank of PDX models. (**A**) RNA sequencing data from ER+, HER2 amplified, and triple-negative PDX tumors were combined with TCGA transcriptomic data. PAM50 subtyping of PDX samples was performed, and a subset of the PAM50 gene set was used in hierarchical clustering of the combined dataset. (**B**) Oncoplot depicting mutated genes, amplifications, and RNA fusions in cell line and PDX models of ER+, HER2 amplified, and triple-negative breast cancer. (**C**) Gene signature score calculation was performed, and a selection of GSEA gene signatures was used in hierarchical clustering of ER+ PDX models.

Genomic and transcriptomic analysis was performed to evaluate the intrinsic PAM50 subtypes and mutational landscape of a bank of ER+, HER2-amplified, and TNBC PDXs and cell line models. First, bulk RNA sequencing was performed and integrated with The Cancer Genome Atlas (TCGA) transcriptomic data to demonstrate all 20 PDX models were grouped within the expected subtype and distributed throughout the TCGA data (Figure 2A). The predicted pathogenicity of missense, frameshift, nonsense, and fusion mutations, as well as CNV, splice variants, and the introduction of stop codons in all PDX models and 14 cell lines, was established using the targeted mutational profiling tool Oncomine v3 (Figure 2B) [23]. The most common mutations were found in TP53, with the second most common being PIK3CA, which has been previously reported for many of these models [49]. The ESR1, ARID1A, and FGFR1 mutations were represented within the ER+ PDX samples. Additionally, we discovered that BCM-15057 expressed the ESR1-CCDC170 fusion mutant, which is associated with a poor response to endocrine therapy, and has not previously been reported, to the best of our knowledge [50]. Additionally, BCM-15034 exhibits a copy number variation of 5.54 of ERBB2, the gene that codes for the HER2 protein (Figure 2B, Supplementary Figure S2B). Bulk RNA sequencing data from five ER+ PDX models were used to generate gene signature scores for a selection of gene sets (Figure 2C). The hierarchical clustering of these signatures demonstrated the heterogeneity within the ER+ models. In all, the PDX models in this study were representative of the heterogeneity of human disease with regards to mutational landscape, intrinsic subtyping, and gene expression.

### 3.3. Genes Associated with Proliferation and ER Signaling Were Profiled at the Single-Cell Level in ER+ PDX Models

**Figure 3:**
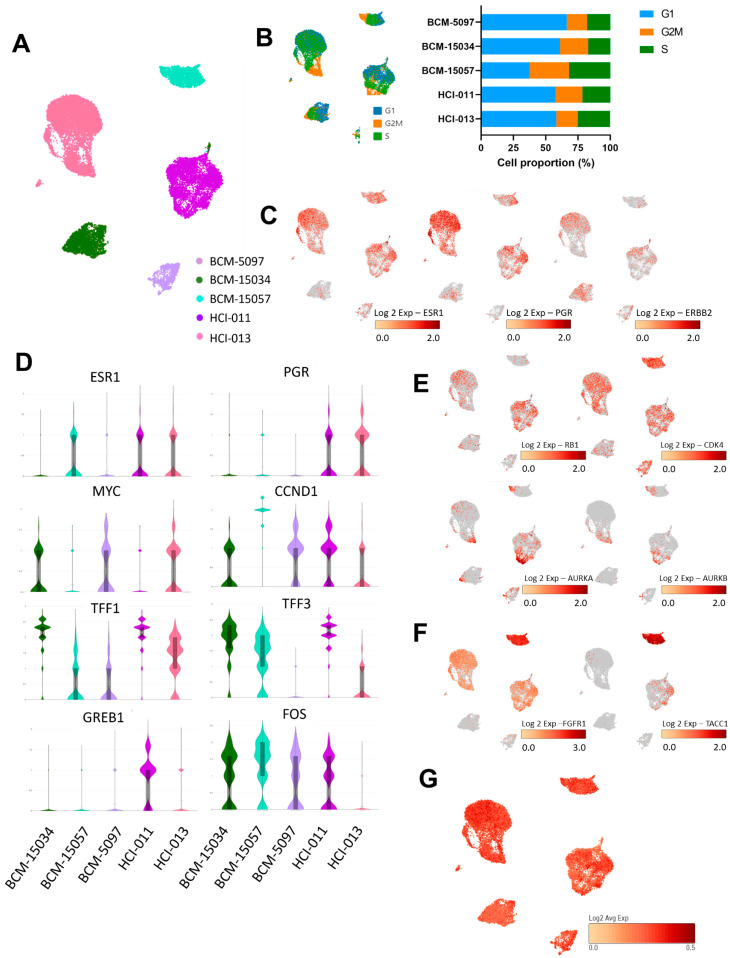
Single-cell transcriptomic analysis of ER signaling in 5 ER+ breast cancer PDX models. (**A**) UMAP of cells from 5 ER+ orthotopic PDX models. Colors represent each model. (**B**) Phases of the cell cycle overlayed onto the UMAP of each of the PDX models and the quantification of percentage of each model in the phases of the cell cycle. (**C**) ESR1, PGR, and ERBB2 expression in respective populations. (**D**) Violin plots representing expression of ESR1 and target genes of ER. (**E**) Cell-cycle-related transcript expression in ER+ PDX models. (**F**) FGFR1 and TACC1 expression in all models, particularly in BCM-15057. (**G**) Gatza_2017_overexpressed_PIK3CA signature score for single cells from 5 ER+ PDX models.

Single-cell transcriptomic profiling was performed to establish transcriptome patterns within the ER+ PDX models. After processing, 21,499 cells from the PDX mammary tumors across the five samples were merged, which displayed clusters unique to each model (Figure 3A,B). Each model expressed ESR1, PGR, and ERBB2 to varying degrees, which corresponded to established protein product expression (Figure 1A and Figure 3C). ER-target gene expression also varied among models, with FOS being the most ubiquitously expressed transcript under estrogen-stimulated conditions, and HCI-011 expressing the most robust ER-target gene signature (Figure 3D). Cell-cycle-associated genes RB1 and cyclin-dependent and aurora kinases were most expressed in BCM-15057, HCI-011, and HCI-013 (Figure 3E), corresponding to the tumor growth rate (Figure 1B), as well as to the proportion of cells in the G2M/S phase in each model (Figure 3B). In addition to an amplification of FGFR1 within BCM-15057, which was revealed by Oncomine, transcriptional upregulation of TACC1 was also present (Figure 3F). Interestingly, TACC1 can form a complex with aurora kinase A, which controls translation and cell division in breast cancer [51]. It has been established that HCI-011 tumors harbor a pathogenic mutation to PIK3CA. Here, we demonstrate that HCI-011 displayed a high gene signature score for overexpressed PIK3CA. Clinically, tumors that express pathogenic PIK3CA mutations are often treated with targeted therapy; however, BYL-719 combination therapy exhibited additive to antagonistic effects in HCI-011 in vitro (Figure 3G) [23].

### 3.4. Tamoxifen Treatment Upregulated Genes Associated with Hormone Therapy Resistance

**Figure 4:**
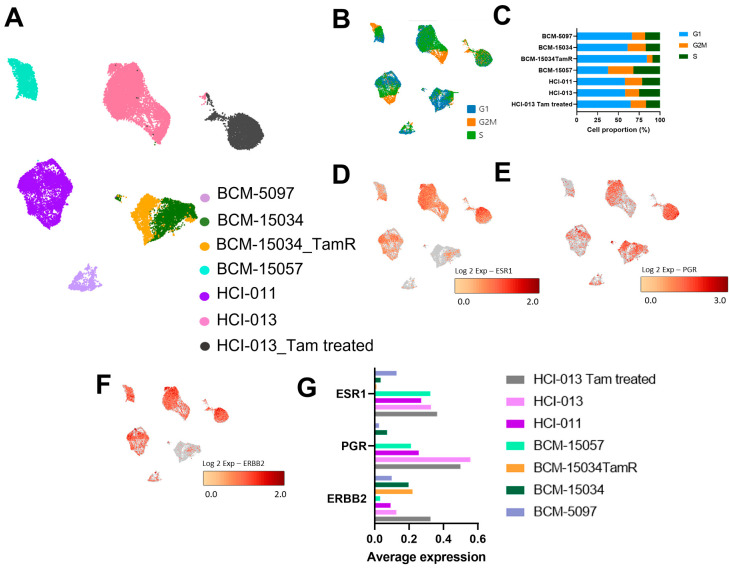
Establishment of transcriptomic changes induced by tamoxifen treatment or resistance at single-cell resolution. (**A**) UMAP of PDX cells that are either untreated, tamoxifen-treated, or tamoxifen-resistant (TamR). (**B**) UMAP, as in A, depicting cells in phases of the cell cycle. (**C**) Respective percentage of each PDX in G1, G2M or S phase of the cell cycle. (**D**–**F**) ESR1, PGR, and ERBB2 expression in 5 ER+ PDX models, as well as one tamoxifen-treated model, and one tamoxifen-resistant model. (**G**) Average expression of ESR1, PGR, and ERBB2 in untreated and tamoxifen-treated PDX tumor cells.

**Figure 5:**
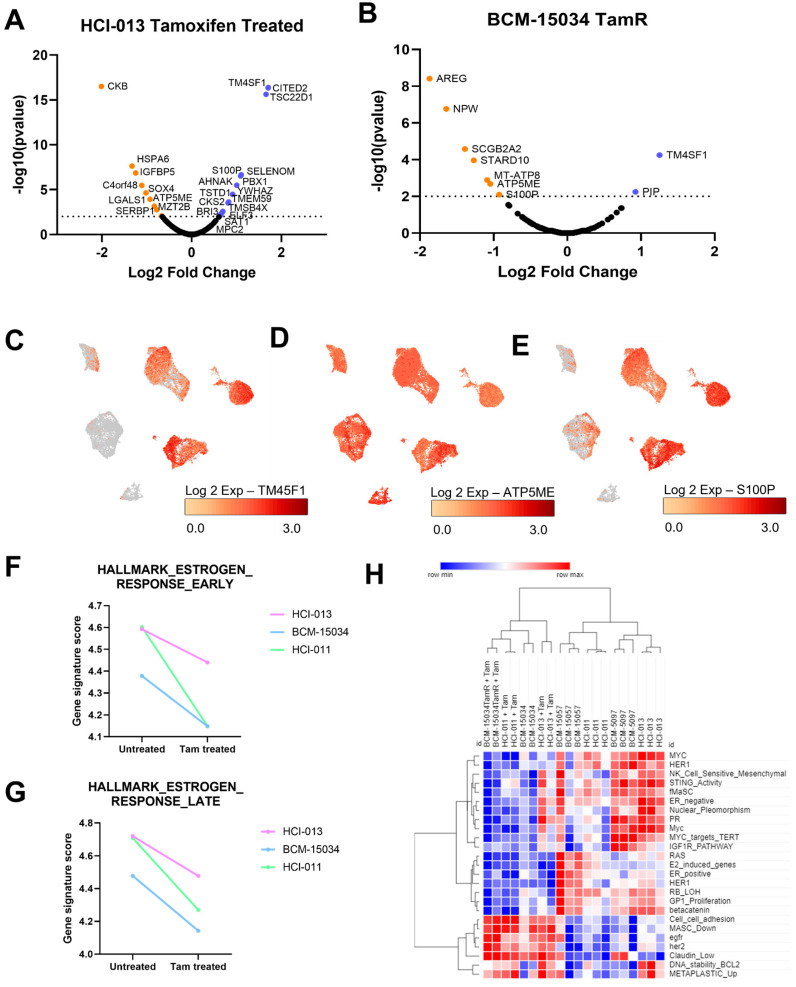
Single-cell and bulk transcriptomic profiling of tamoxifen-treated PDX models. (**A**,**B**) Volcano plots of differentially expressed genes in tamoxifen-treated HCI-013 and BCM-15034TamR. (**C**–**E**) UMAP of genes that are differentially expressed in both tamoxifen-treated and TamR conditions. (**F**,**G**) Line plot comparison of estrogen response early (**F**) and estrogen response late (**G**) gene signatures in bulk RNA sequencing from 3 different ER+ PDX models. (**H**) Heatmap of unsupervised clustering of selected gene signatures from bulk RNA-seq data of tamoxifen-treated and untreated PDX tumors.

We next sought to evaluate changes induced by tamoxifen treatment by comparing tamoxifen-treated HCI-013 with untreated cells, as well as BCM-15034 and an isogenic BCM-15034 that has become tamoxifen resistant (BCM-15034TamR). In both cases, tamoxifen reduced the average expression of PR, and increased ERBB2 transcript expression (Figure 4). A comparison of genes that were differentially expressed between the HCI-013 tamoxifen-treated and untreated groups showed increased expression of transcriptional regulators (CITED2, TSC22D1, and PBX1) and decreased expression of genes associated with responses to cAMP and translation (SERBP1, IGFBP5, CKB) (Figure 5A). While the majority of transcripts were expressed at a lower level in BCM-15034TamR when compared to the parental line, only two genes were expressed at a higher level: PIP and TM4SF1 (Figure 5A,B). Interestingly, only three genes were differentially regulated following treatment in both models. TM4SF1 transcripts were increased in both BCM-15034TamR and HCI-013 following treatment, and ATP5ME was expressed at a lower level in both models. However, S100P was more highly expressed in tamoxifen-treated HCI-013 when compared to the untreated PDX and was expressed less in BCM-15034TamR (Figure 5C–E). Regardless of the differences in gene expression, gene signature analysis showed lower early and late estrogen response gene signature scores in tamoxifen-treated cells, as expected (Figure 5F–G). Additionally, gene signatures that show the greatest difference in tamoxifen-treated groups included lower scores for proliferative and pro-survival signatures and higher scores in EGFR- and HER2-associated signatures (Figure 5H). These results validated the previously established upregulation of EGFR and HER2, which occurs as a response to the downregulation of estrogen signaling and can be associated with acquisition of an endocrine-therapy-resistant phenotype [52].

### 3.5. HTS Identified VX-680 and Simeprevir as Additively Effective Compounds with Tamoxifen

**Figure 6:**
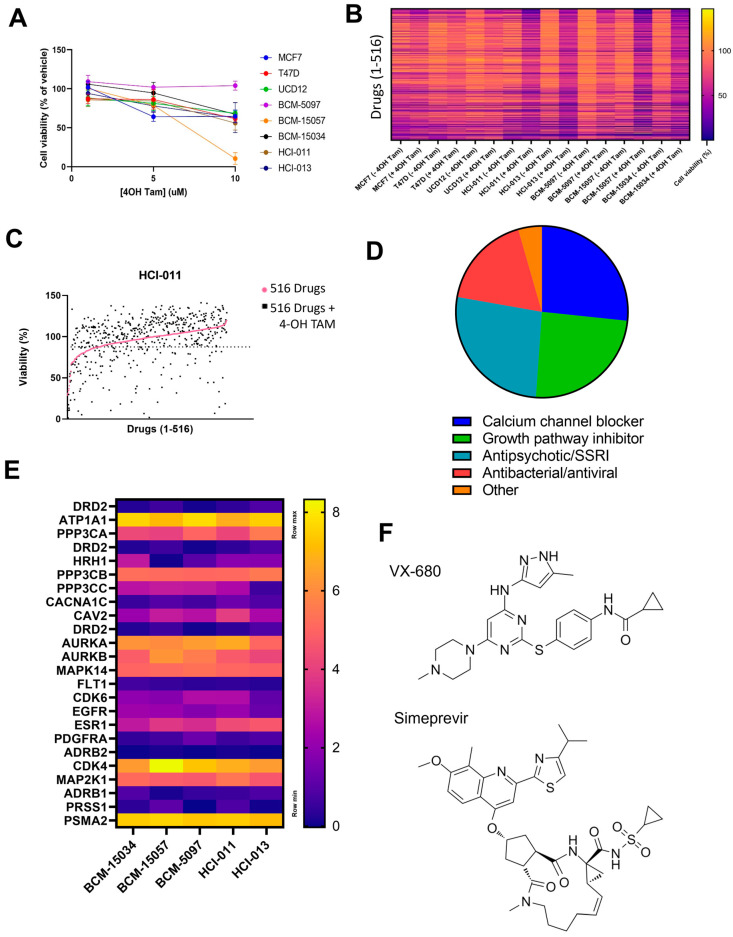
Identification of tamoxifen synergistic compounds. (**A**) Cell viability of 3 cell lines and 5 PDX models when treated with 4-OH tamoxifen in vitro. (**B**) A heatmap representing the cell viability from HTS of 3 cell lines and 5 ER+ PDX models using a 516-drug library alone, or in combination with 4-OH tamoxifen. (**C**) Representative graph showing the additivity of drugs with 4-OH tamoxifen in HCI-011, with a dotted line to represent viability of cell treated with 4-OH alone. (**D**) Pie chart showing the different classes of the 45 drugs that were found to be additive in the 5 ER+ PDX models. (**E**) Heatmap representing log2TPM expression of drug targets identified in (**D**) from bulk RNA sequencing of PDX mammary tumor cells. (**F**) Chemical structure of VX-680 and simeprevir.

We next sought to discover novel combinatorial agents to be administered with tamoxifen as a more durable form of treatment. First, PDX suspension cultures were screened using 4-OH tamoxifen (4-OH TAM) alone to establish a dose that decreases viability to roughly 90%. While all models remained sensitive to tamoxifen metabolites in vitro, BCM-5097 remained insensitive (Figure 1B and Figure 6A). HTS with a library of 516 single agents alone or in combination with 4-OH TAM was performed on 3 ER+ cell lines and 5 PDX suspension culture models to determine which compounds reduced cell viability below that which could be accounted for by either compound as a single agent (Figure 6B,C). Forty-five combinations were found to be additive in all models based on the coefficient of drug interaction (CDI), with the exception of BCM-5097 which was excluded from analysis due to being insensitive to tamoxifen alone (Figure 6D). All known targets of possible agents were evaluated at the RNA level (Figure 6E). Aurora kinase A and B transcripts were highly expressed in all models, as were ATP1A1 and CKD4. However, some targets of the most synergistic compounds are currently unknown in the context of breast cancer and were therefore excluded from this analysis. After excluding compounds that were contraindicated with tamoxifen, the two most additive combinations, an aurora kinase inhibitor, VX-680, and a Hepatitis C antiviral compound, simeprevir, were selected for further study (Figure 6F) [53,54].

### 3.6. Tamoxifen Combination Therapy Was Effective in PDX Models of ER+ Breast Cancer

**Figure 7:**
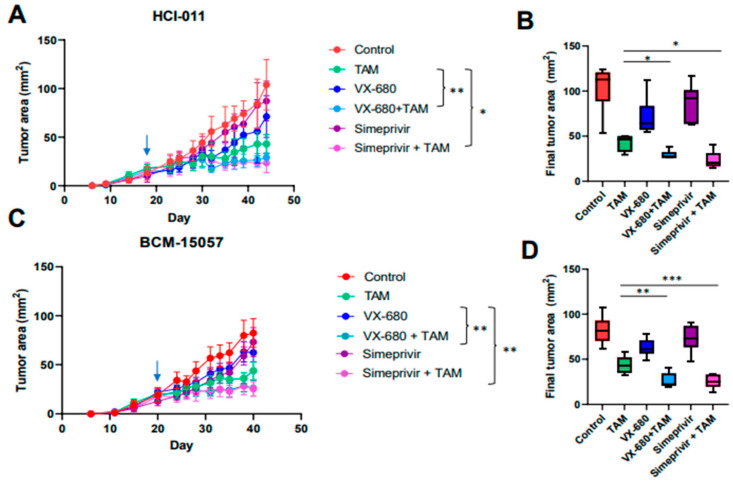
In vivo testing of selection tamoxifen combination therapy. (**A**,**C**) In vivo growth curve of orthotopic mammary tumors and final tumor area of HCI-011 (**A**) and BCM-15057 (**C**). Arrows denote the start of treatment. Two-way ANOVA with Tukey’s multiple comparison was performed where * *p* < 0.05 and ** *p* = 0.005. (**B**,**D**) Box and whisker plots of final tumor area of each group in HCI-011 (**B**) and BCM-15057 (**D**). An unpaired *t*-test was performed to compare the tamoxifen-treated group with VX-680 + TAM and simeprevir + TAM individually. * *p* < 0.05, ** *p* < 0.005, *** *p* < 0.001.

**Figure 8:**
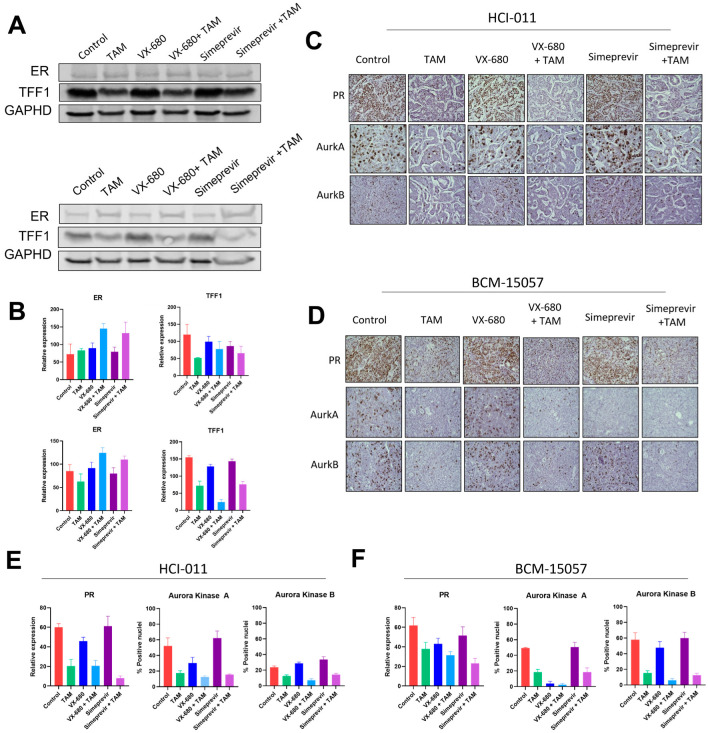
Proteomic analysis of mammary tumors treated with tamoxifen combination therapy. (**A**) Western blot image detecting ER and TFF1 in HCI-011 (top) and BCM-15057 (bottom) mammary tumors following tamoxifen combination treatment, and quantification of densitometry of the HCI-011 (top) and BCM-15057 (bottom) blots is shown in (**B**). The uncropped blots are shown in Figure S3. Representative images of IHC and quantification of PR, aurora kinase A, and aurora kinase B are shown for HCI-011 (**C**,**E**) and BCM-15057 (**D**,**F**). Images of IHC have been taken at 400× magnification.

To evaluate tamoxifen and simeprevir or VX-680 combinations in vivo, BCM-15057 and HCI-011 were chosen based on their moderate sensitivity to tamoxifen (Figure 1B). BCM-15034 was excluded from this study as it expressed high levels of HER2 and would be treated with HER2-targeting therapeutics in a clinical setting. In both models, drug combination groups showed a profound reduction in tumor volume when compared to vehicle-treated control mice, as well as a significantly better reduction than the groups treated with either single agent or tamoxifen alone (Figure 7A–D). ER, PR, and TFF1 levels were evaluated as indications that tamoxifen treatment effectively downregulated ER signaling. As expected, ER target genes TFF1 and PR were downregulated, and ER levels were slightly increased (Figure 8A–D, Supplementary Figures S3–S5). Additionally, aurora kinase A and B protein expression was analyzed and reduced expression was seen following treatment with VX-680 (Figure 8C–F). Both combinations were effective in reducing tumor volume beyond that of tamoxifen as a mono-agent or either simeprevir or VX-680 alone, indicating that combination therapy could provide a more durable treatment for patients with ER+ breast cancer.

### 3.7. Tamoxifen Combination Therapy Abrogated Tamoxifen-Treatment-Induced Upregulation of HER2

**Figure 9:**
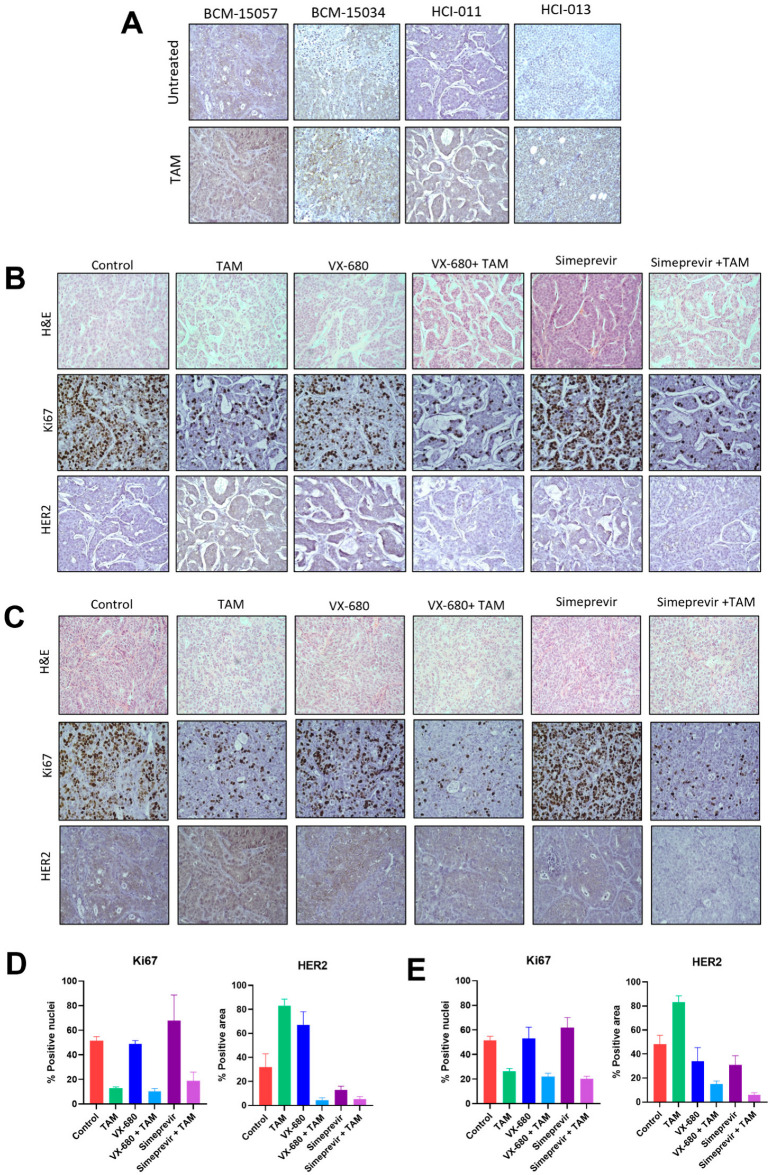
Immunohistochemistry of PDX tumors treated with tamoxifen or combination therapy. (**A**) HER2 IHC staining of 5 orthotopic ER+ PDX models with and without treatment with tamoxifen in vivo. (**B**) H&E, Ki67, and HER2 staining of HCI-011 mammary tumors from mice treated with tamoxifen combination therapies, and the quantification of positive nuclei or positive area (**D**). (**C**) H&E, Ki67, and HER2 staining of BCM-15057 mammary tumors from mice treated with tamoxifen combination therapies, and the quantification of positive nuclei or positive area (**E**). All images of IHC have been taken at 400× magnification.

Endocrine therapy resistance with respect to tamoxifen has been widely studied. It has been established that estrogen signaling downregulates HER2 expression, which can be reversed by antiestrogens such as tamoxifen [55]. This upregulation of HER2 can drive tamoxifen resistance by facilitating ER-independent proliferative and pro-survival signaling. We established that expression of HER2 is increased in all tamoxifen-sensitive models following tamoxifen treatment (Figure 9A, Supplementary Figure S6). Interestingly, when BCM-15057 and HCI-011 were treated with tamoxifen and simeprevir or VX-680 combination therapy, HER2 was not upregulated to the same degree as in tumors treated with tamoxifen alone (Figure 9B–D, Supplementary Figure S7). This suggests that antiestrogen combination therapies should be pursued that mitigate endocrine therapy resistance mechanisms as a more durable form of treatment.

## 4. Discussion

Although endocrine therapy has been utilized for the treatment of ER+ breast cancer for decades, as many as 40% of patients will suffer local or distant recurrence, with many cases acquiring endocrine therapy resistance [56]. More durable treatment options may include combination therapy with antiestrogens to prolong remission-free survival and decrease the incidence of endocrine therapy resistance. In this study, we identified two different tamoxifen combination regimens, simeprevir and VX-680, that eliminate the upregulation of HER2, which is often induced by tamoxifen treatment and can facilitate endocrine therapy resistance. Clinically, simeprevir or VX-680 may be added to an existing tamoxifen treatment regimen if HER2-dependent progression occurs. Additionally, this combination may be useful in instances of AI resistance, as evidenced by both HCI-011 and BCM-15057 being sensitive to tamoxifen combination therapy in vivo, even though both models were generated from samples taken from patients who had progressed on AI treatment.

The mechanism by which simeprevir and tamoxifen combination therapy leads to a reduced tumor burden, and reduced HER2 expression, is currently unknown, and may necessitate further study. Additionally, many studies have shown the benefit of aurora kinase inhibitors, as well as other cell cycle inhibitors, in combination with antiestrogens [57,58,59]. Although both tamoxifen and aurora kinase inhibitors are cytostatic, to our knowledge, this is the first study to identify the additional benefit of reduction in HER2 upregulation provided by combination therapy when compared to tamoxifen monotherapy. This may account for the improved anti-tumor activity of this particular combination. Additionally, while this study utilized tamoxifen combination therapies administered concomitantly, it remains unknown if these compounds may provide benefit if prescribed in series, or if pre-treatment with tamoxifen affects the reduction in tumor burden and HER2 expression. These gaps in knowledge may be the subject of further studies. Nevertheless, the present study demonstrates the need for antiestrogen combinations to suppress known mechanisms of endocrine resistance to potentiate their activity.

While tamoxifen has improved patient outcomes by means of overall survival and recurrence-free survival, 30% of patients ultimately progress on tamoxifen therapy or shortly after cessation [60,61,62]. Although this has been established for many years, it is recently that the US FDA has approved the first widely utilized tamoxifen combination therapy [63]. Abemaciclib with tamoxifen was approved for the treatment of ER+ breast cancer with high risk of recurrence in 2021, and as of 2023 has been expanded to include a larger patient population. Therefore, establishing any downstream effects on known mechanisms of endocrine-therapy resistance induced by abemaciclib–tamoxifen combination therapy may also be beneficial.

In this study, we sought to develop novel therapeutic combinations with tamoxifen to enhance anti-tumor efficacy with the hope of developing more durable treatments for ER+ breast cancer. We identified two compounds, simeprevir and VX-680, through HTS that were able to reduce tumor volume in vivo more than either compound or tamoxifen alone. Additionally, tumor cells from the combination groups did not show upregulation of HER2, which is a hallmark of tamoxifen treatment. Our results demonstrate the importance of developing combinations that address the potential mechanisms of endocrine therapy resistance as a more durable form of treatment for ER+ breast cancer patients.

## 5. Conclusions

The present study identifies two novel tamoxifen combination therapies that increase the efficacy of tamoxifen in vivo. The results reported herein establish the importance of pursuing combinations that reduce the expression or activation of known resistance mechanisms, which can provide more durable treatment in the clinical setting.

## Data Availability

The scRNA seq data presented in this manuscript are available online. The data have been deposited on GEO; accession numbers are listed in the Supplementary Materials.

## Supplementary Materials

The following supporting information can be downloaded at: https://www.mdpi.com/article/10.3390/cancers15123179/s1, Figure S1: Representative histograms of signal intensity of IHC images corresponding to Figure 1A; Figure S2: (A) Heatmap representing patient ER, PR and HER2 status from which PDX models were generated, as reported by the Baylor College of Medicine PDX Portal. (B) Copy number of HER2 in PDX model BCM-15034 as established by Oncomine; Figure S3: Complete western blot images of in vivo experiments included in Figure 8; Figure S4: Representative histograms of signal intensity of IHC images corresponding to Figure 8C; Figure S5: Representative histograms of signal intensity of IHC images corresponding to Figure 8D; Figure S6: Representative histograms of signal intensity of IHC images corresponding to Figure 9A; Figure S7: Representative histograms of signal intensity of IHC images corresponding to Figure 9B,C; Table S1: Bulk and scRNA samples used in this study.
