# Supplementary material for: Stratification of Tamoxifen Synergistic Combinations for the Treatment of ER+ Breast Cancer
Source: Cancers (Basel). 2023 Jun 14;15(12):3179. doi: 10.3390/cancers15123179 (PMC10296623; doi:10.3390/cancers15123179)
Supplement: Supplementary file 1 [file cancers-15-03179-s001.zip › cancers-2403040-supplementary.pdf]

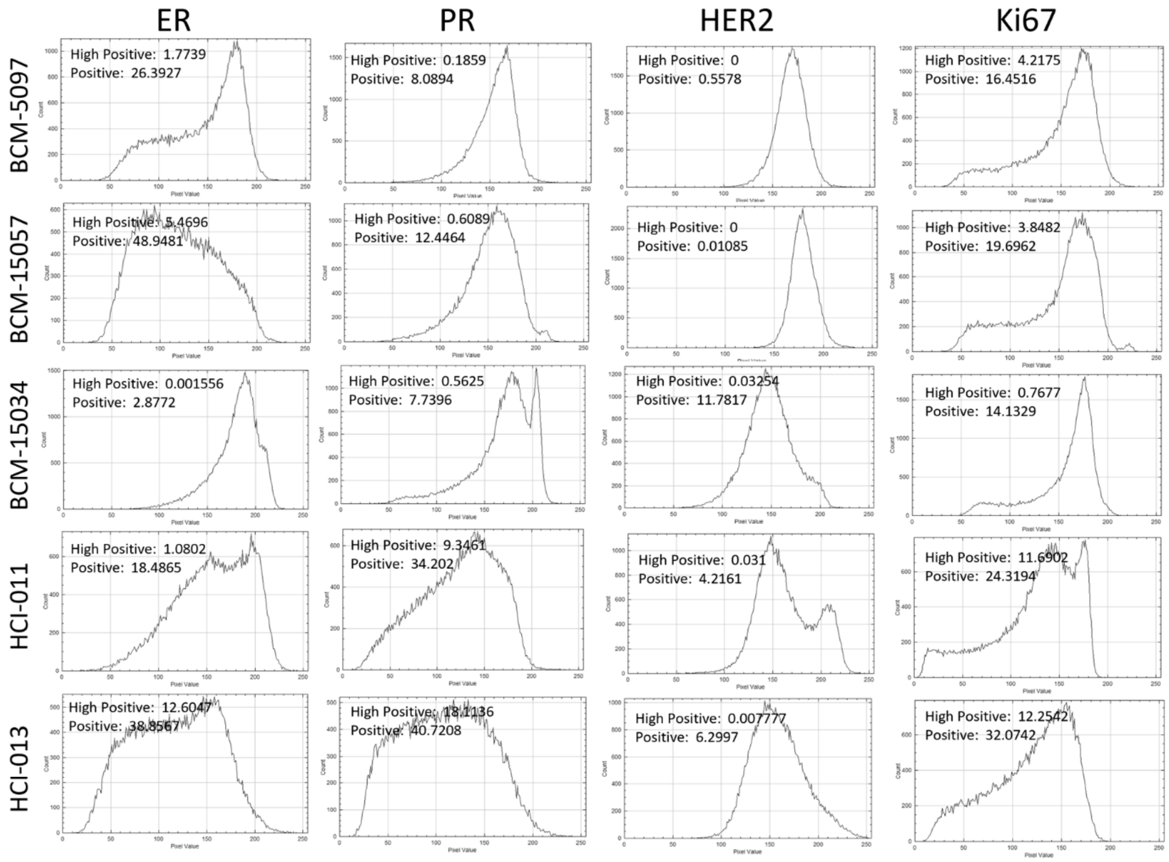

Supplemental Figure S1. Representative histograms of signal intensity of IHC images corresponding to Figure 1A. High positive and positive scores are listed as a percentage of pixels.

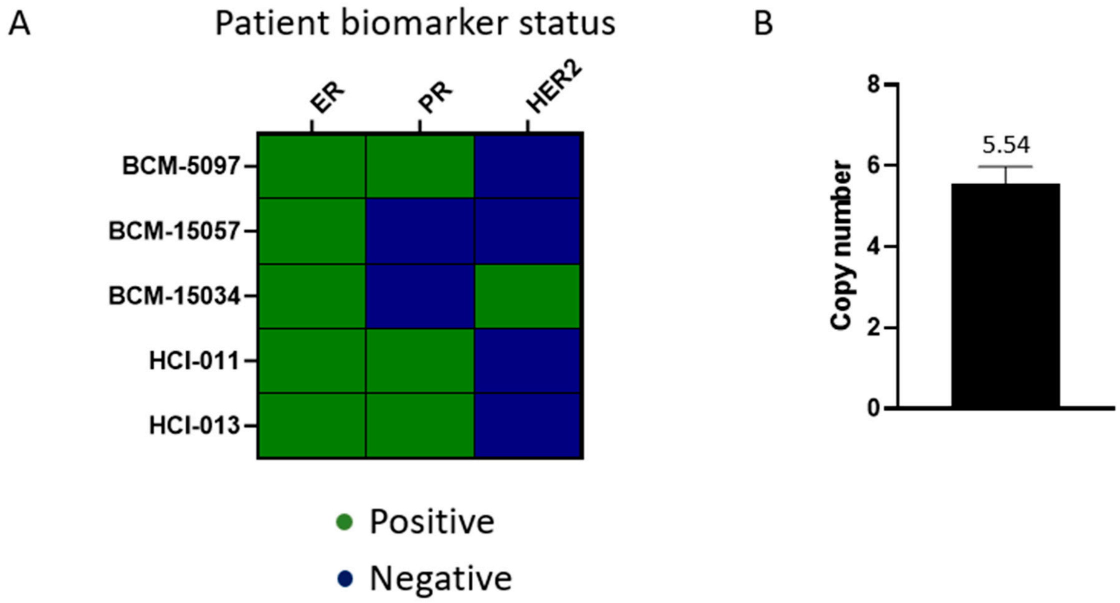

Supplemental Figure S2. (A) Heatmap representing patient ER, PR and HER2 status from which PDX models were generated, as reported by the Baylor College of Medicine PDX Portal. (B) Copy number of HER2 in PDX model BCM-15034 as established by Oncomine.

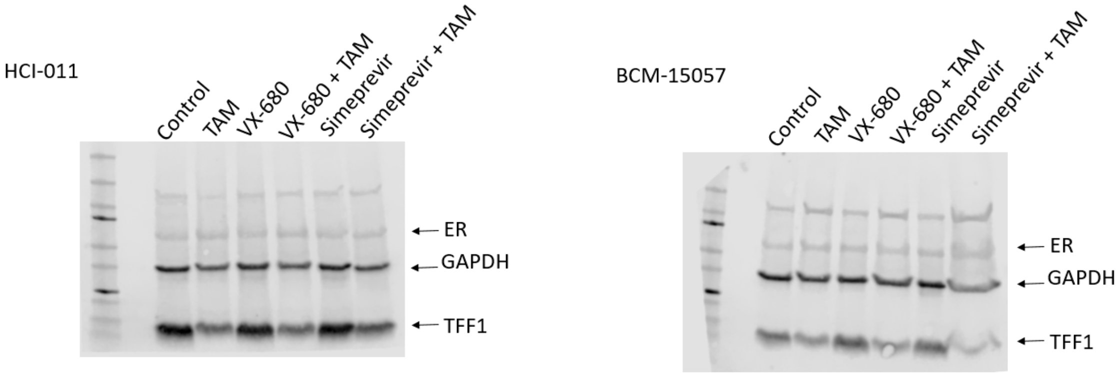

Supplemental Figure S3. Complete western blot images of in vivo experiments included in Figure 8

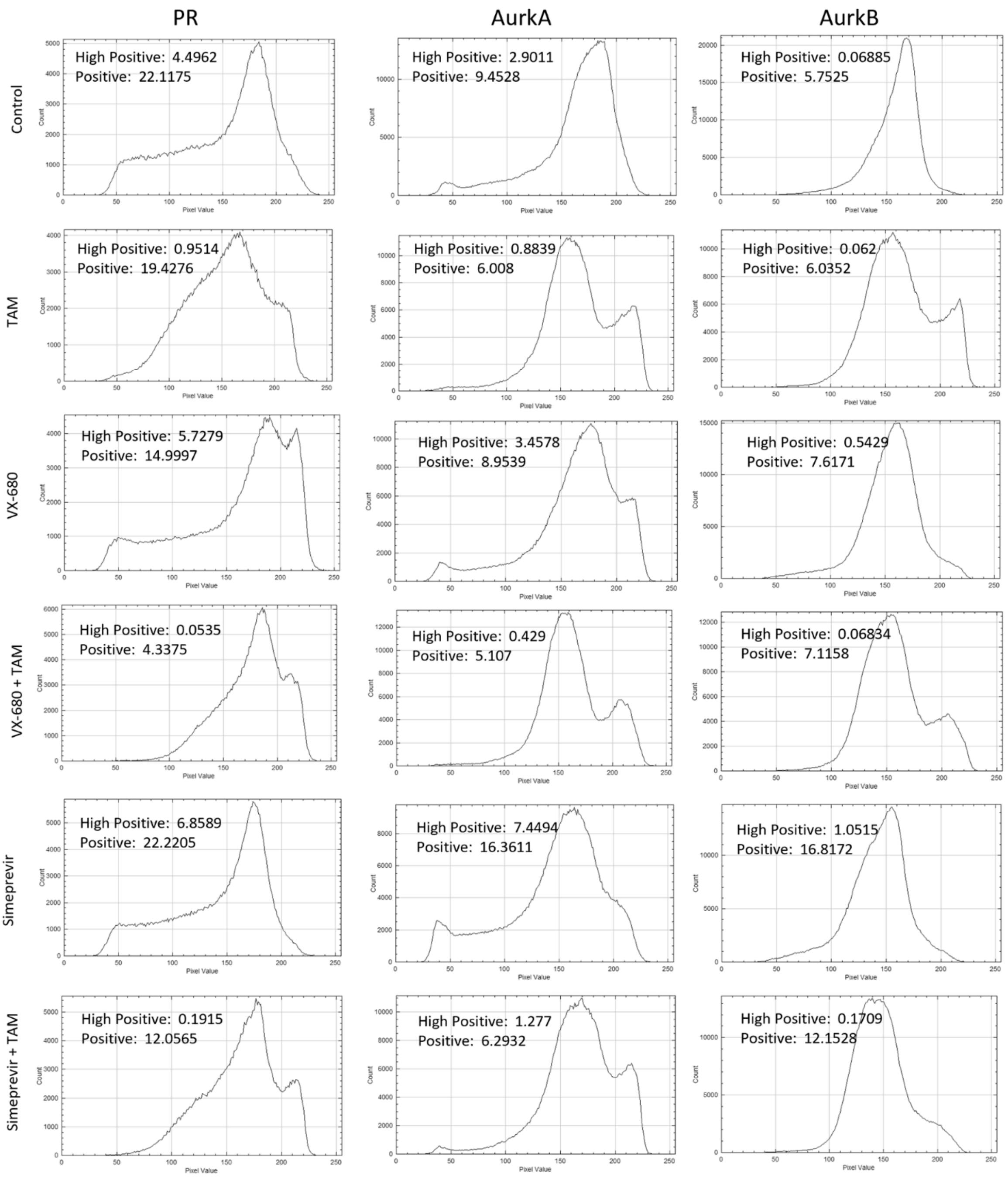

Supplemental Figure S4. Representative histograms of signal intensity of IHC images corresponding to Figure 8C. High positive and positive scores are listed as a percentage of pixels.

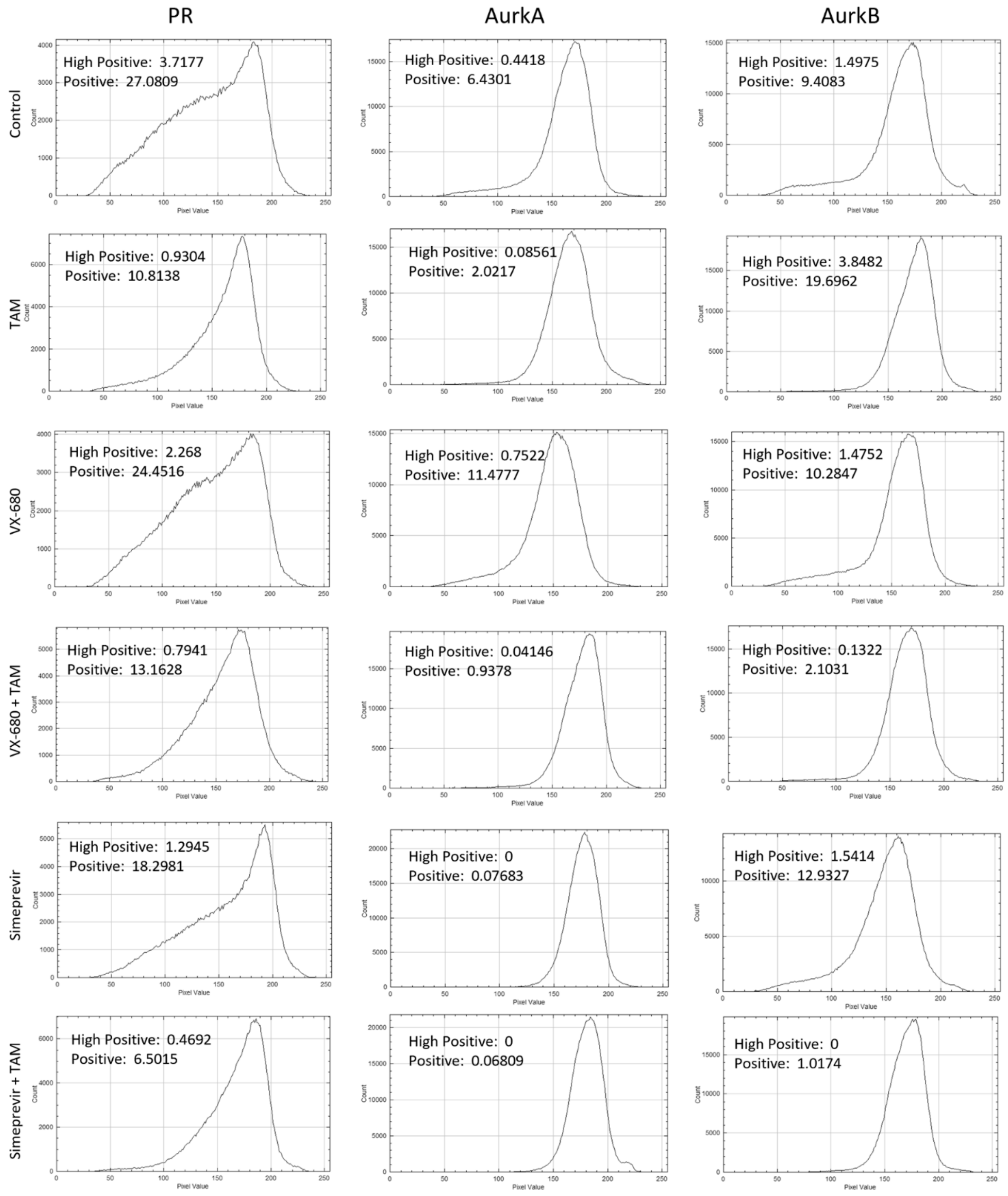

Supplemental Figure S5. Representative histograms of signal intensity of IHC images corresponding to Figure 8D. High positive and positive scores are listed as a percentage of pixels.

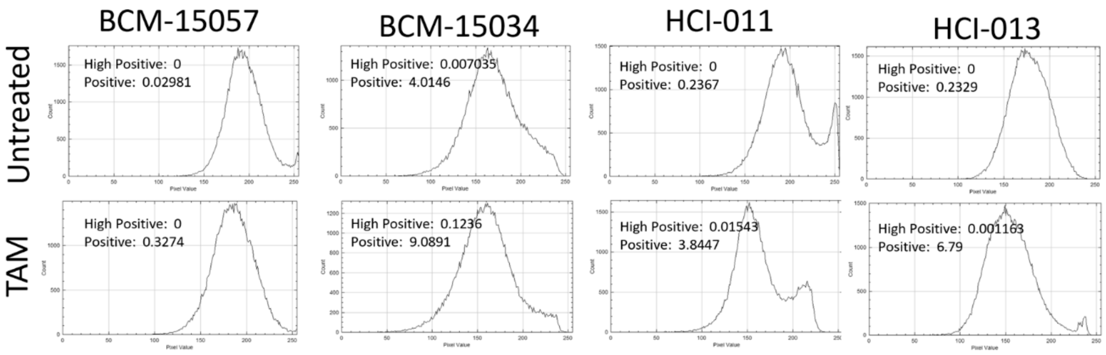

Supplemental Figure S6. Representative histograms of signal intensity of IHC images corresponding to Figure 9A. High positive and positive scores are listed as a percentage of pixels.

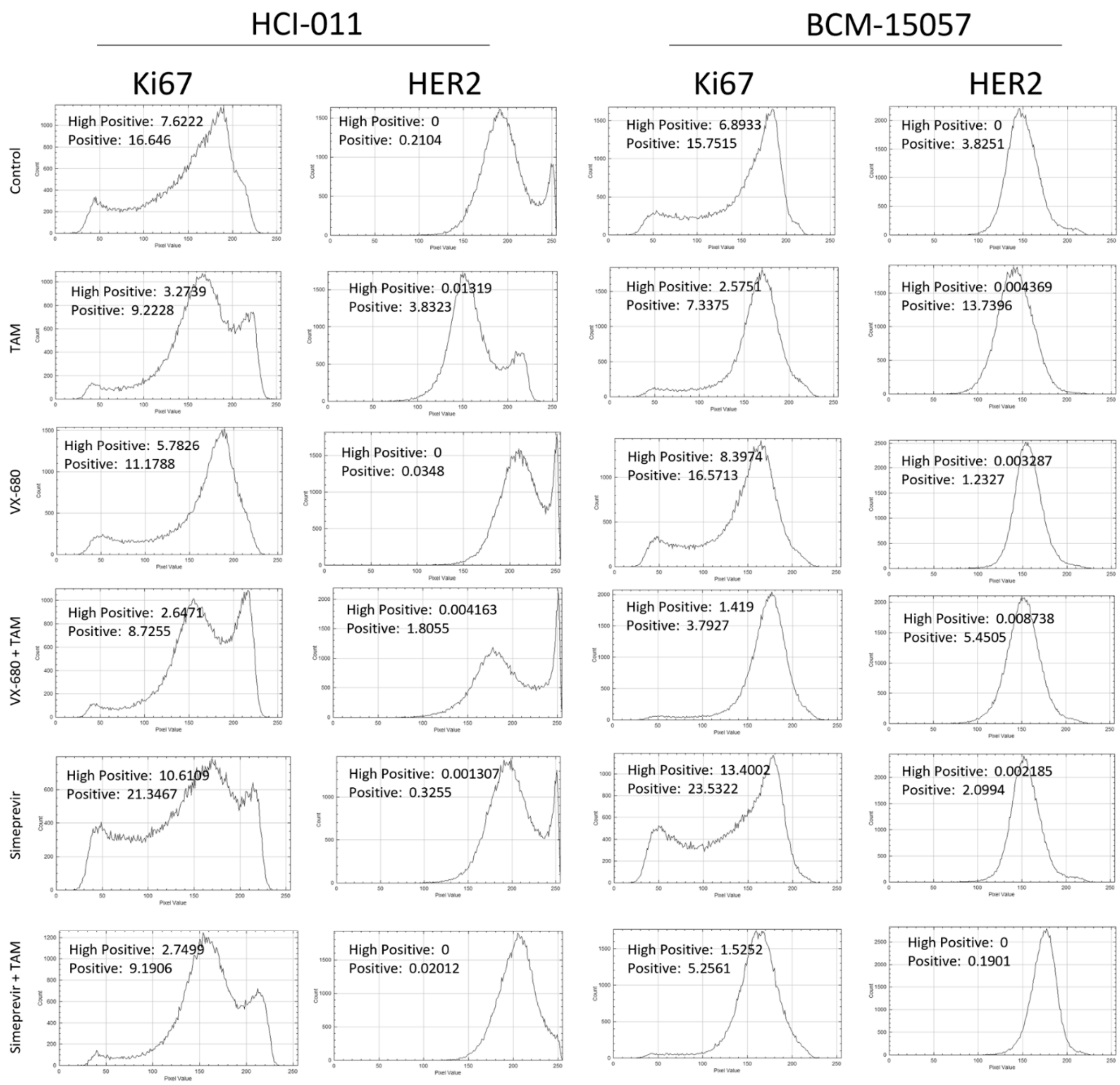

Supplemental Figure S7. Representative histograms of signal intensity of IHC images corresponding to Figure 9B-C. High positive and positive scores are listed as a percentage of pixels.

Supplemental information

GSE	Samples (bulk)		GSE	Samples (single cell)
GSE118942	HCI01.MGT.101020<br>HCI01.MGT.101021<br>HCI01.MGT.101022<br>HCI02.MGT.100945<br>HCI02.MGT.100946<br>HCI02.MGT.100948<br>HCI03.MGT.102031<br>HCI04.MGT.100763<br>HCI08.MGT.100879<br>HCI09.MGT.100241<br>HCI09.MGT.100508<br>HCI09.MGT.100511<br>HCI09.MGT.100512<br>HCI10.MGT.100276<br>HCI10.MGT.100277	HCI13.MGT.100822<br>PT52.MGT.100302<br>PT52.MGT.100303<br>PT52.MGT.100304<br>PT52.MGT.100775<br>PT52.MGT.100916<br>PT52.MGT.100917<br>WHIM2.MGT.100000<br>WHIM2.MGT.100027<br>WHIM2.MGT.100076<br>WHIM30.MGT.100431<br>WHIM30.MGT.175278A<br>WHIM30.MGT.181040A<br>WHIM30.MGT.182696A<br>WHIM30.MGT.NSG21A	In progress	BCM-15034_107352<br>BCM-15034_107244<br>BCM-15034TamR_107634<br>BCM-15057_107684<br>BCM-5097_107348<br>HCI-011_107322<br>HCI-011_107409<br>HCI-013_106662<br>HCI-013_106663<br>HCI-013_107785
GSE189325	WHIM2.107647<br>WHIM2.107647b	WHIM2.107668<br>WHIM2.107672		
In progress	BCM.0132.107564<br>BCM.15034.107894<br>BCM.15057.107957<br>BCM.15057.107968<br>BCM.2147.107741<br>BCM.2147.107744<br>BCM.2147.107745<br>BCM.2147CR2.107841<br>BCM.2147CR2.107842<br>BCM.2277.107573<br>BCM.3887.107696<br>BCM.5097.107568<br>BCM.5097.107931<br>HCI.001.107935<br>HCI.011.107898<br>HCI.013.107857<br>HCI.013.107939<br>UCD52CR21.107961<br>UCD52CR21.107964<br>UCD52CR21.107966<br>UCD52CR21.107967<br>WHIM30.107947	WHIM30CR6.107919<br>WHIM30CR6.107920<br>WHIM30CR7.107949<br>WHIM30CR7.107950<br>WHIM30CR7.107951<br>BCM.15034.107510<br>BCM.15057.107336<br>BCM.5097.107567<br>BCM.7482.107504<br>HCI.001.100097<br>HCI.011.107518<br>HCI.011.107522<br>HCI.013.102249<br>UCD52.100398<br>UCD52.100916<br>UCD52.107627<br>UCD52CR.107532<br>UCD52CR.107591<br>UCD52CR.107592<br>WHIM30.107661<br>WHIM30.107662<br>WHIM30CR.107502		
In progress	HCI.013.107415<br>BCM.15034TamR3.107922<br>BCM.15034TamR3.107924	BCM.15057EI3.107630<br>BCM.15057EI3.107640		

Supplemental Table S1. Bulk and scRNA samples used in this study.

Drugs included in high throughput screening		
Gatifloxacin	Salmeterol xinafoate	CX-4945 (Silmitasertib)
Gemcitabine HCl	Saquinavir mesylate	Bleomycin Sulfate
Rigosertib (ON-01910,Estybon)	Simeprevir	Linsitinib
Fulvestrant	Tamibarotene	BYL-719 (Alpelisib)
Cilnidipine	Teneligliptin hydrobromide	Pazopanib Hydrochloride
Lidocaine	TH-302	LY2157299
Dexrazoxane HCl (ICRF-187, ADR-529)	Vinblastine sulfate	VX-809
Epothilone B (EPO906, Patupilone)	Vinorelbine ditartrate	Alendronate sodium
Celecoxib	Ziprasidone	Arbidol HCl
Enzastaurin (LY317615)	Ziprasidone hydrochloride monohydrate	Chlorprothixene
Aprepitant	Doxorubicin	Clotrimazole
Posaconazole	Lapatinib Ditosylate	Conivaptan HCl
IPI-145 (INK1197)	MLN2238	Cytarabine
Ramelteon	CEP-18770	Dinaciclib (SCH727965)
Tenofovir Disoproxil Fumarate	Disulfiram	Diphenylpyraline HCl
Vincristine	Marimastat	Docosanol (Abreua)
Entecavir Hydrate	17-AAG (KOS953)	Dovitinib Dilactic acid
Vatalanib (PTK787) 2HCl	AT13387 (Onalespib)	Duloxetine HCl
LY2835219	Elvitegravir (GS-9137)	Emtricitabine
Mianserin HCl	Rocilinostat (ACY-1215)	Enoxacin (Penetrex)
Imatinib Mesylate (STI571)	Vorinostat (SAHA, MK0683)	Estradiol
Doxorubicin (Adriamycin) HCl	CUDC-101	Fenticonazole Nitrate
Pelitinib (EKB-569)	Pracinostat (SB939)	Furosemide
Cediranib (AZD217)	Belinostat (PXD101)	Gabapentin
5-Azacytidine	PCI-24781 (CRA-024781)	Gemcitabine
Macitentan	MLN8237 (Alisertib)	Indomethacin
Carfilzomib (PR-171)	VX-680 (MK-0457,Tozasertib)	Lamivudine
MEK162 (ARRY-162, ARRY-438162)	Barasertib (AZD1152-HQPA)	Lithocholic Acid
Etoposide	Birinapant (TL32711)	Meloxicam (Mobic)
Bendamustine HCl	YM155	Oxethazaine
Plerixafor (AMD3100)	Amuvatinib (MP-470, HPK 56)	Pitavastatin Calcium
Saracatinib (AZD0530)	Ketoconazole	Prochlorperazine
Bosutinib (SKI-606)	Sildenafil Citrate	Rosuvastatin Calcium
Carboplatin	Mycophenolate Mofetil	Simvastatin (Zocor)
Lenvatinib (E7080)		Terfenadine
Cladribine		Thioridazine HCl
Tivozanib (AV-951)		Vitamin D2 (Ergocalciferol)
Mercaptopurine (6-MP)		GSK2126458 (Omipalisib)
Floxuridine		BI6727 (Volasertib)
Acitretin		Chloroquine diphosphate
Fluoxetine HCl		Deferasirox

Epirubicin HCl<br>Pamidronate Disodium<br>Idarubicin HCl<br>Malotilate<br>Ampicillin<br>Kanamycin Sulfate<br>Tetracycline Hydrochloride<br>Vortioxetine (Lu AA21004) HBr<br>Carmofur<br>Fluvoxamine maleate<br>Aprotinin<br>Nafamostat Mesylate(FUT-175)<br>Amonafide<br>Ivermectin<br>Doxazosin Mesylate<br>Nepafenac<br>Masitinib (AB1010)<br>Loratadine<br>Foretinib (GSK1363089)<br>Cabozantinib (XL184, BMS-907351)<br>PCI-32765 (Ibrutinib)<br>CAL-101 (Idelalisib, GS-1101)<br>ABT-263 (Navitoclax)<br>Sorafenib<br>Dasatinib (BMS-354825)<br>(R)-Crizotinib<br>Pazopanib (GW-786034)<br>Afatinib dimaleate<br>Bardoxolone methyl<br>Boceprevir<br>Cariprazine<br>Cinacalcet<br>CO-1686 (AVL-301)<br>Cobimetinib<br>Colchicine<br>Dabigatran etexilate mesylate<br>Docetaxel Trihydrate<br>Dronedarone<br>Elacridar<br>Eltrombopag Olamine<br>EMD-1214063<br>Erlotinib<br>Fluvastatin	Aminophylline<br>Methotrexate<br>Pralatrexate<br>Pyrimethamine<br>Fluvastatin Sodium<br>Lovastatin<br>Atorvastatin Calcium<br>Evacetrapib (LY2484595)<br>Lonafarnib<br>Tolcapone<br>Ganetespib (STA-9090)<br>Pemetrexed<br>Paclitaxel (Taxol)<br>Docetaxel<br>10-DAB (10-Deacetylbaccatin)<br>Mitomycin C<br>Streptozocin<br>Melphalan<br>Asenapine<br>Ivacaftor (VX-770)<br>Heparin sodium<br>Ondansetron HCl<br>Alfuzosin HCl<br>Linezolid<br>Clopidogrel<br>Prazosin HCl<br>TSU-68 (SU6668,Orantinib)<br>Ispinesib (SB-715992)<br>Ziprasidone HCl<br>LY2228820<br><br>Abiraterone acetate<br>Ritonavir<br>Lopinavir<br>Atazanavir<br>AZD6244 (Selumetinib)<br>Lapatinib<br>Gefitinib (ZD1839)<br>Nilotinib(AMN-107)	Sunitinib<br>Ulipristal acetate<br>Doripenem<br>Cytarabine hydrochloride<br>AZD-9291 (Osimertinib)<br>Efavirenz<br>Nelfinavir<br>Pitavastatin<br>Ondansetron hydrochloride dihydrate<br>Meropenem trihydrate<br>(+)-Ketoconazole<br>Haloperidol hydrochloride<br>Valproic acid<br>Glycopyrrolate<br>Reserpine hydrochloride<br>Carvedilol<br>Nebivolol<br>Epinephrine Bitartrate<br>Miltefosine<br>Phenformin HCl<br>Spironolactone<br>Ciclopirox ethanolamine<br>Amiodarone HCl<br>Loperamide HCl<br>Tamoxifen Citrate<br>Trifluoperazine 2HCl<br>Benidipine HCl<br>Amlodipine<br>Amlodipine Besylate<br>Flunarizine 2HCl<br>Cinacalcet HCl<br>Rimonabant<br>Acemetacin<br>Etodolac<br>Tolfenamic Acid<br>Procarbazine HCl<br>Chlorpromazine HCl<br>Dopamine HCl<br>Clomiphene citrate<br>Mifepristone<br>Toremifene Citrate<br>Raloxifene HCl

Ledipasvir<br>Luliconazole<br>Mefloquine hydrochloride<br>Micafungin sodium<br>MK-4305<br>Nelfinavir Mesylate<br>Pemetrexed disodium hemipenta hydrate<br>Perampanel<br>Pregabalin<br>Regorafenib hydrochloride<br>RG7388<br>Salirasib<br>Benzydamine HCl<br>Bosentan<br>Broxyquinoline<br>Cetrimonium Bromide (CTAB)<br>Cetylpyridinium Chloride<br>Chlorocresol<br>Chlorquinaldol<br>Chlorzoxazone<br>Clindamycin Phosphate<br>Clofazimine<br>Closantel<br>Closantel Sodium<br>Dirithromycin<br>Domiphen Bromide<br>Ebastine<br>Epinastine HCl<br>Ethacridine lactate monohydrate<br>Fidaxomicin<br>Furaltadone HCl<br>Lomerizine HCl<br>Loxapine Succinate<br>Mechlorethamine HCl<br>Mevastatin<br>Neomycin sulfate<br>Nifuroxazide<br>Oxymetholone<br>Pimecrolimus<br>Piperacillin Sodium<br>Primaquine Diphosphate<br>Proflavine Hemisulfate	Erlotinib Hydrochloride<br>Regorafenib<br>Sorafenib Tosylate<br>Afatinib (BIBW2992)<br>Nintedanib (BIBF 1120)<br>Sunitinib malate<br>Crenolanib (CP-868596)<br><br>Quinapril HCl<br>Adefovir Dipivoxil<br>Palonosetron HCl<br>Amitriptyline HCl<br>Clomipramine HCl<br>Clozapine<br>Olanzapine<br>Paroxetine HCl<br>Prucalopride<br>Sertraline HCl<br>Dapoxetine HCl<br>LDE225 (NVP-LDE225,Erismodegib)<br>Propafenone HCl<br>Daunorubicin HCl<br>Irinotecan HCl Trihydrate<br>Topotecan HCl<br>Apatinib<br>Chlorotrianisene<br>Tamoxifen<br>Cyclobenzaprine HCl<br>Motolimod (VTX-2337)<br>INCB-024360<br>Tacrine hydrochloride<br>Estropipate<br>Puromycin dihydrochloride<br>Digoxin<br>Pimavanserin<br>Brucine<br>Nicotinamide<br>Caffeic acid	Clemastine Fumarate<br>Azelastine HCl<br>Otilonium Bromide<br>Pyridostigmine Bromide<br>Solifenacin succinate<br>Tolterodine tartrate<br>Fosbretabulin (Combretastatin A4 Phosphate (CA4P)) Disodium<br>Racecadotril<br>Alverine Citrate<br>Amfenac Sodium Monohydrate<br>Azithromycin Dihydrate<br>Benzbromarone<br>Benzethonium Chloride<br>Pimasertib (AS-703026)<br>Daclatasvir (BMS-790052)<br>SAR245409 (XL765)<br>BIRB 796 (Doramapimod)<br>Fasudil (HA-1077) HCl<br>Quizartinib (AC220)<br>Dapagliflozin<br>Cyclosporine<br>Irbesartan<br>Rapamycin (Sirolimus)<br>Everolimus (RAD001)<br>DAPT (GSI-IX)<br>Amprenavir (agenerase)<br>Temsirolimus<br>Dacomitinib (PF299804, PF299)<br>Cisplatin<br>Tivantinib (ARQ 197)<br>LDK378

Sertaconazole nitrate	Cytisine	Eltrombopag
Sodium ascorbate	Artemether	Ticlopidine HCl
Sodium butyrate	Chlorogenic acid	Ticagrelor
Sodium Nitrite	Vinorelbine	Zinc Pyrithione
Thiamphenicol	vinblastine	Candesartan Cilexetil
Tiratricol	Cepharanthine	Cyproheptadine hydrochloride
Valganciclovir HCl	CP-945598 HCl	Dibucaine
Valnemulin HCl	Dovitinib (TKI-258, CHIR-258)	Ciclesonide
Azathioprine	Altretamine	Tiagabine
Bisacodyl	Linifanib (ABT-869)	Bedaquiline fumarate
Bromhexine HCl	PSI-7977	Bedaquiline
Butoconazole nitrate	Sildenafil	Chlorhexidine digluconate
Chenodeoxycholic Acid	Artemether (SM-224)	Promethazine HCl
Chloroxine	Cobicistat (GS-9350)	Losmapimod
Crystal Violet	Imiquimod	Mupirocin
Cyclamic acid	Ponatinib (AP24534)	Hydroxychloroquine Sulfate
Cyclosporin A	ABT-199	Lomitapide
Diclazuril	Embelin	Desogestrel
Diclofenac Diethylamine	Neratinib (HKI-272)	Octreotide acetate
Diphenhydramine HCl	Orotic acid	Pentamidine isethionate
Dronedarone HCl	Anidulafungin	Topotecan
Econazole nitrate	EMPTY	Pentobarbital sodium salt
Erythromycin Ethylsuccinate	Cabozantinib malate (XL184)	Radotinib(IY-5511)
Isoconazole nitrate	Azelnidipine	Pexidartinib (PLX3397)
Isoniazid	Licofelone	Entrectinib
Levosimendan	KPT-330	R788 disodium
Menadione	Mizoribine	Doxycycline HCl
Mequinol	Benserazide HCl	L-Glutamine
Miconazole	Bazedoxifene HCl	Fingolimod (FTY720)
Miconazole Nitrate	Mubritinib (TAK 165)	Vandetanib (ZD6474)
Mycophenolic acid	Tripelennamine HCl	TOK-001
Nadifloxacin	Rupatadine Fumarate	Oxaliplatin
Nalidixic acid	Homatropine Methylbromide	Pacritinib
Nedaplatin	Amoxapine	VRT752271
Nifedipine	Bacitracin	
Nisoldipine	Capreomycin Sulfate	
Oxytetracycline (Terramycin)	Chlorhexidine HCl	
Rifampin	Colistin Sulfate	
Sodium salicylate	Diacerein	
Sorbitol	Flubendazole	
Streptomycin sulfate		
Sulbactam sodium		
Sulconazole Nitrate		

Sulfamerazine<br>Sulfameter<br>Sulfamethizole<br>Terbinafine HCl<br>Tilmicosin<br>Tioconazole<br>Toltrazuril<br>Zafirlukast<br>Diethylstilbestrol<br>D-Mannitol<br>Doxercalciferol<br>Doxifluridine<br>Haloperidol<br>Idebenone<br>Itraconazole<br>Mitoxantrone HCl<br>Rifapentine<br>Calcitriol<br>Tacrolimus (FK506)<br>Fosaprepitant dimeglumine salt<br>Sitafloxacin Hydrate<br>Alfacalcidol<br>Cabazitaxel<br>Poziotinib<br>Pimozide<br>Ciclopirox<br>Oltipraz<br>Triclosan	Fudosteine<br>Meclizine 2HCl<br>Minocycline HCl<br>Nicardipine HCl<br>Alendronate<br>Naratriptan<br>K-115<br>DL-α-Difluoromethylornithine (hydrochloride hydrate)<br>Atovaquone<br>Fostamatinib (R788)<br>Darunavir Ethanolate<br>SKF 525A (hydrochloride)<br>BAF312 (Siponimod)<br>Gallic acid<br>Maprotiline HCl<br>Trimipramine (maleate)<br>Napabucasin<br>Theophylline<br>Aminoguanidine hydrochloride<br>Imipramine (hydrochloride)	
